# Active tunable plasmonically induced polarization conversion in the THz regime

**DOI:** 10.1038/srep34994

**Published:** 2016-10-13

**Authors:** Furi Ling, Gang Yao, Jianquan Yao

**Affiliations:** 1 Wuhan National Laboratory for Optoelectronics, Huazhong University of Science and Technology, Wuhan, Hubei 430074, China; 2 School of Optical and Electronic Information, Huazhong University of Science and Technology, Wuhan, Hubei 430074, China; 3 College of Precision Instrument and Opto-electronics Engineering, Tianjin University, Tianjin 300072, China

## Abstract

A plasmon-induced polarization conversion (PIPC) structure based on periodically patterned graphene was demonstrated in the THz regime. By varying the Fermi level of two connected T-shape graphene strips through the electrostatic gating, the peak frequency and the group index in the transparency window can be tuned, which is good agreement with the coupled Lorentz oscillator model. Due to interference between two polarization selective graphene plasmonic resonances coexisting in the planar metamaterial, polarization conversion can be achieved. The linearly polarized THz wave can be converted to elliptically and right circularly polarized THz wave through varying the relaxation time of electrons in graphene. This novel chip-scale active terahertz device promises essential application opportunities in terahertz sensing and terahertz communications.

In recent years, as a concept extended by electromagnetically induced transparency (EIT), plasmon-induced transparency (PIT) mimicked by metamaterial has attracted tremendous interests^1^. Compared to the quantum EIT effect restricted to the extreme experiment condition, the pronounced PIT phenomenon can be emulated in metamaterials via destructive interference between the radiative and non-radiative plasmonic modes or by breaking the symmetry of the metamaterial system. Therefore, PIT based on metamaterials has plenty of potential applications in sensing^2^, enhancing nonlinear effect^3^, optical switching and slowing light^4^. And, PIT based on metamaterial structures including cut wire^5^, split-ring resonators^6^,^7^, and coupled waveguide resonators^8^ have been demonstrated both theoretically and experimentally. For practical applications, dynamic tunability of PIT has emerged which relies on integrating metamaterials with optical active materials such as liquid crystals, semiconductors^9^,^10^, and graphene^11^. Among all of them, the metamaterials based on graphene have been presented and investigated in the terahertz region^3^,^12^. And it is undergoing a period of rapid development and diversified applications in the imaging^13^, sensing^14^, and communications^15^,^16^. Now, the applications of PIT based on metamaterials not only demand highly efficient group index and amplitude modulation but also highly sensitive terahertz polarizers.

Recently, it was also discovered that metamaterials are ideal structures to convert the linear THz wave to cross-linear THz wave^17^, and to convert the linear THz wave to elliptical polarization^18^,^19^. Due to strong light-matter interactions at the quantum level^20^ and the Fermi energy controlled through doping or electrostatic gating^21^, active tunable polarization conversions based on graphene have been presented and researched on theory in the mid-IR and terahertz frequencies^22^,^23^. To active control the wavefront of electromagnetic waves, dynamically tunable anomalous refraction based on graphene nanocrosses has been achieved in the infrared regime^24^. A metadevice by integrating a single layer of graphene with an anisotropic metasurface has been reported in the infrared regime, which can realize polarization encoding and the PDM (polarization-division multiplexing) technique^25^. Therefore, designing a dynamically tunable polarization converter based on graphene remains an ongoing interest in the terahertz regime, especially for the voltage control, which is one of the simplest ways among all tunable techniques^23^.

In this letter, a dynamically tunable PIPC based on the periodically patterned graphene, consisting of two graphene dipole resonance and two graphene monopole resonance, has been demonstrated in the THz region. From the simulation results, one can see that the peak frequency and the group index in the transparency window can be tuned by varying the Fermi energy of two connected T-shape graphene strips through controlling the voltage of the electrostatic gating. Meanwhile, the PIT based on graphene can convert linear THz wave to elliptical polarization. Furthermore, the elliptical polarization can be varied to right circular polarization through controlling the relaxation time of electron in graphene, which offers possible application as a controllable polarization converter.

## Results

**Figure 1:**
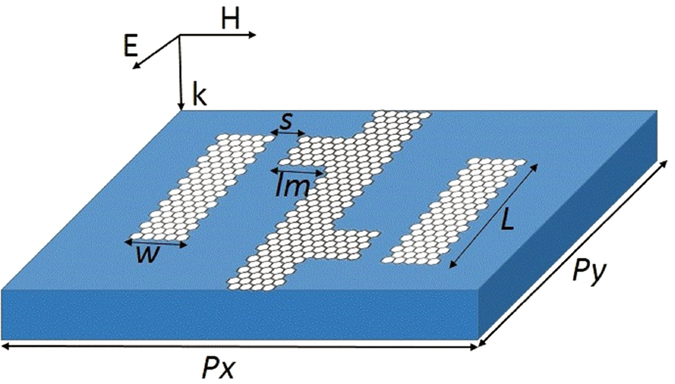
Schematic view of PIPC unit structure consisting of two grapheme cut wire and two connected T-shape graphene strips. Schematic model of the designed PIPC unit cell with the *Px* = *10* *um*, *Py* = *8* *um*, *L* = *6* *um*, *lm* = *2* *um, s* = *0.3* *um*.

**Figure 2:**
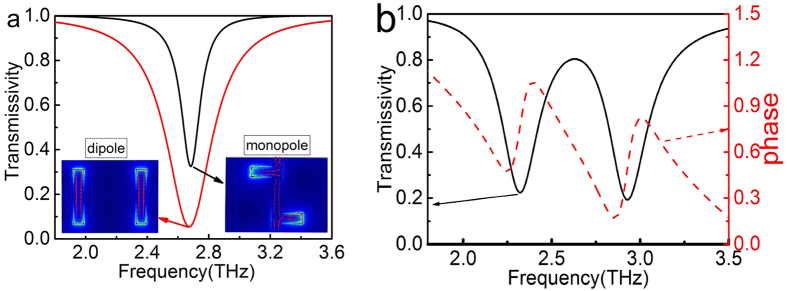
(**a**) Simulated transmissivity spectra of two disconnected vertical graphene strips (black line) and two connected T-shape graphene strips (red line), the inset show the electric displacement of dipole resonance (left) and monopole resonance (right), (**b**) power transmittance (black line), phase spectra of the PIPC (red line).

A schematic of the proposed PIPC is shown in the Fig. 1, with its geometrical parameters described in the caption. COMSOL Multiphysics is used to optimize the PIPC structure, with the periodic boundary conditions in *x–y* plane. A single unit cell contains two graphene dipole resonance and two graphene monopole resonance^26^ placed on the substrate of silicon dioxide. The *y*-polarized THz wave propagating along +*z* direction impinge into the PIPC. Meanwhile, the power transmittance can be displayed as 
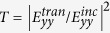
, where 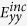 is the electric field of the *y*-polarized incident THz wave and 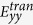 is the electric field of the *y*-polarized transmitted THz wave, respectively. According to the simulation results, a dipole resonance at 2.6 THz is obtained by the directly radiation coupling for a unit cell only containing two disconnected vertical graphene strips, with the incident electric field polarization along to *y*-axis. As the electric field polarization rotates to the *x*-axis, a monopolar resonance at 2.6 THz can also be achieved for a unit cell only containing two connected T-shape graphene strips, as shown in the Fig. 2(a) with the red line. And the corresponding electric field distribution and surface current are shown in the inset.

**Figure 3:**
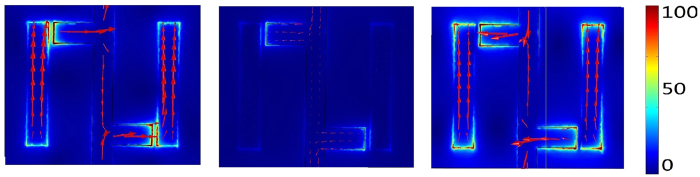
Electric field distribution (color) and surface current distribution (red arrows) for the frequency (**a**) 2.3 THz, (**b**) 2.6 THz, (**c**) 2.9 THz.

Then, the vertical graphene strip with two attacched horizontal graphene strips and two disconnected vertical graphene strips are placed in proximity, as shown in the Fig. 1. When the incident electric field is parallel to *y*-axis, the incident THz wave allows the excitation of the dipole resonance in the disconnected vertical graphene strip. The charge on the top of the vertical graphene strip induced by the incident *y* polarized electric field will induce the charge of an opposite sign on the tip of the horizontal graphene strip, thus resulting in the excitation of the monopole resonance. Then, the graphene monopole resonance in turn interacts with the graphene dipole resonance and suppresses its resonance in a narrow frequency range by destructive interference. Hence, a transparency window with peak frequency at 2.6 THz and two enhanced power transmittance dips at 2.3 THz and 2.9 THz are observed, as shown in the Fig. 2(b) as the black line. From the corresponding phase spectra, as shown in the Fig. 2(b) as the red line, the steep dispersion between the two transmissivity dips can be observed. The electric field and the surface current distributions of the two enhanced transmissivity dips have been calculated to better understand the physical process involved. It can be found that the current of two vertical graphene dipole and two connected T-shape graphene monopole are in-phase at frequency 2.3 THz, as shown in the Fig. 3(a), resulting from the radiative elements directly excited by the incident THz wave. While the current of two vertical graphene dipole and two connected T-shape graphene monopole are anti-phase at frequency 2.9 THz^27^, as shown in the Fig. 3(c). In contrast, the nearly complete suppression of graphene monopole resonance leads to a near zero electric field intensity in the disconnected vertical graphene strip at the peak frequency in the transparency window, as shown in the Fig. 3(b).

**Figure 4:**
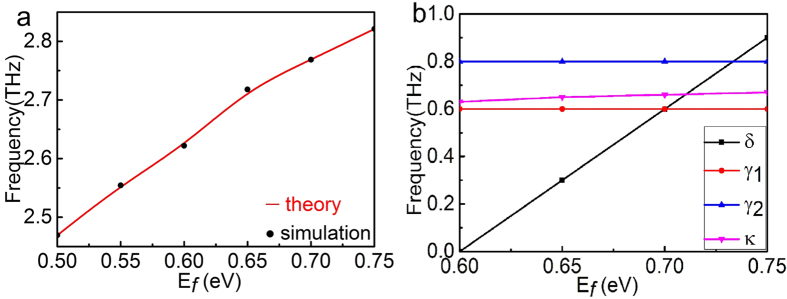
(**a**) The peak frequency in the transparency window as a function of *E*_*f*_ with the black points corresponding to the simulated result and the red line corresponding to the analysis result. (**b**) Extracted values of *δ*, *γ*_1_, *γ*_2_, *κ* as a function of the Fermi level *E*_*f*_ according to the (**a**).

The ability to dynamically tune the peak frequency over a broad frequency range by controlling the Fermi energy levels of two connected T-shape graphene strips is one of the remarkable characteristic for the PIPC structure. Figure 4(a) shows the peak frequency in the transparency window as a function of the Fermi energy level of two connected T-shape graphene strips. The number of charge carriers contributed to the graphene monopole oscillation increases with the Fermi level of two connected T-shape graphene strips change from 0.5 eV to 0.6 eV. Thus, the graphene monopole resonance frequency first shifts to the graphene dipole resonance frequency, corresponding to 2.6 THz with the Fermi level of two vertical graphene strips maintaining at 0.6 eV. Then, the difference between the graphene monopole resonance frequency and the graphene dipole resonance frequency becomes larger with the Fermi level of two connected T-shape graphene strips change from 0.6 eV to 0.75 eV. In this case, the peak frequency in the transparency window is blue-shifted, while the peak amplitude exceeds 75% and spectral line width of the transparency window remains almost unchanged. By combing the resonance condition with surface plasmon satisfied equation 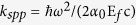, the resonant frequency can be approximately expressed as 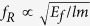, where *α*_0_ is the fine structure constant, and *lm* represents the length of the horizontal graphene strip^28^. The estimate is essentially consistent with the calculated resonant frequencies with different Fermi energies shown in Fig. 4(a). So the transparency window and the power transmittance peak frequency can be tuned by controlling the Fermi level without changing the GPIT (graphene plasmon induced transparency) geometries. It is also can be found that the simulated results is agreed well with the coupled Lorentz oscillator model^5^.

The widely used coupled Lorentz oscillator model is adopted to analyze the near field interaction between graphene strips, leading to better understanding of the physical mechanism of the PIPC behavior. A ground state |0〉, two upper states |1〉 and |2〉 are involved in our model. In analogy to the graphene dipole resonance excited by the incident THz wave in PIPC structure, |0〉 → |1〉 defines the dipole-allowed transition. It is related to the dipole resonance frequency *ω*_0_ and the damping rate *γ*_1_ of the radiative mode. Meanwhile, |0〉 → |2〉 defines the monopole-forbidden transition corresponding to the graphene monopole resonance excited only through coupling with graphene dipole resonance in PIPC structure. And it is characterized by a damping rate *γ*_2_ of non-radiative model. The coupling coefficient *κ* is correlated with coupling between the graphene dipole resonance and the graphene monopole resonance, and *δ* denotes the detuning from the graphene monopole resonance to the graphene dipole resonance. Consequently, the two possible path ways, namely, |0〉 → |1〉 and |0〉 → |1〉 → |2〉→ |1〉 interfere destructively, resulting in a very narrow transparency window within a broader absorption band. To provide a quantitative description of our coupled Lorentz oscillator model, the amplitudes *χ*_1_ and *χ*_2_ in upper states |1〉 and |2〉 can be used to describe this interference, which is shown as^5^,^9^:


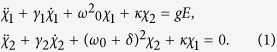


where *g* is a geometric parameter indicating the coupling strength of the radiative plasmonic state with the incident THz wave. By solving the equation (1), the susceptibility of the GPIT metamaterial layer is expressed as 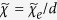 with the susceptibility of one PIPC metamaterial unit cell displayed as 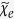 and a thickness of *d*. The near-field susceptibility can be approximately regarded as the far-field power transmittance for the PIPC metamaterial, with the approximation that sufficiently thin PIPC metamaterial layer is thinner than the wavelengths of the incident THz waves. So the far-field power transmittance of the PIPC structure can be regarded as^9^:


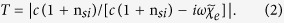


Subsequently, theoretical analysis results are plotted in Fig. 4(a) by red line according to the equation (2) and the simulated results are presented black point for a direct comparison. It is evident that the simulated results are reproduced nearly perfectly by the fitted results, confirming the validity of the designed PIPC graphene device. The fitting values of *δ*, *γ*_1_, *γ*_2_, *κ* as a function of the Fermi level *E*_*f*_ are shown in the Fig. 4(b). The radiative damping rate *γ*_1_ and non-radiative damping rate *γ*_2_ are 0.6 THz and 0.8 THz, respectively. And the coupling coefficient *κ* has a small increasing trend with Fermi level. However, the damping rates *γ*_1_, *γ*_2_ and the coupling strength *κ* do not vary significantly with increasing Fermi energy. This implies that variation in Fermi energy hardly affects the modulation strength, power transmittance peak, and the spectral linewidth of the PIPC effect. The detuning parameter *δ* increases markedly as the Fermi level increases, resulting from the larger resonance frequency difference between the graphene dipole resonance and the graphene monopole resonance. This indicates that the peak frequency in the transparency window can be tuned to lower or higher frequencies by varying the Fermi level.

**Figure 5:**
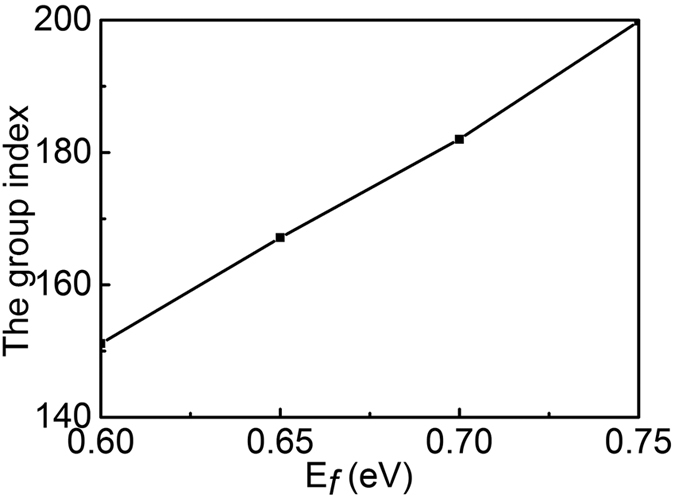
The maximum value of *n*_*g*_ within the transparency window as a function of *E*_*f*_.

Because of the ability to greatly slow down the speed of THz wave for the PIPC structure, and the slow light has potential application in terahertz communication^29^. It is highly desired that the group index *n*_*g*_ of the PIPC structure can be able to actively tune, especially electrical tuning. The group index *n*_*g*_ in the transparency window is obtained according to the relation *n*_*g*_ = *n* + *ωdn*/*dω* with the effective refractive index *n* extracted by using the *S*-parameter retrieval method^30^. The max value of the group index in the transparency window for the grahene PIPC structure at different Fermi level *E*_*f*_ are shown in the Fig. 5. It can be seen that the max value of the *n*_*g*_ can below 150 when *E*_*f*_ is small than 0.6 eV, as shown in the Fig. 5. Furthermore, the max value of *n*_*g*_ gradually increases as the Fermi level of the two connected T-shape graphene strip change from 0.6 eV to 0.75 eV, and the max value can reach up to 200. Hence, active tuning of the group index by controlling of the two connected T-shape graphene strip can be achieved.

**Figure 6:**
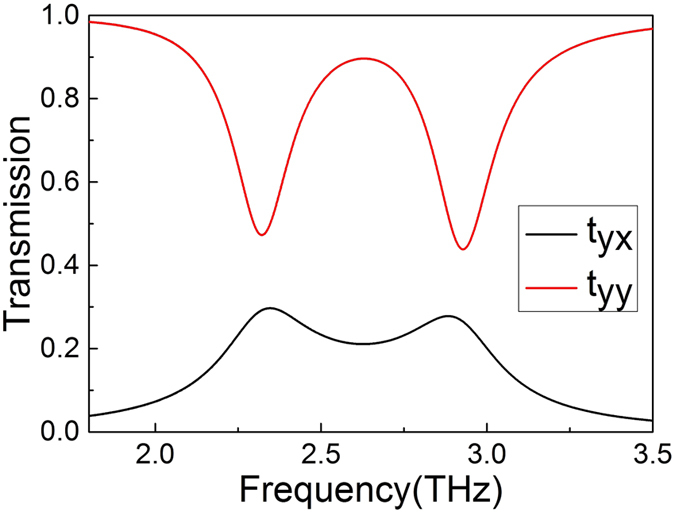
Simulated transmission spectra *t*_*yx*_ and *t*_*yy*_. The red line is the transmission spectra of *t*_*yx*_ and the black line is the transmission spectrax of *t*_*yy*_.

Polarization conversion is another characteristic of the designed PIPC structure. In this case, the transmission coefficient of *y*-polarized and *x*-polarized transmitted waves are defined as 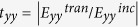 and 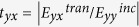, respectively. As one can expected, the graphene monopole resonance excited by the coupling with the graphene dipole resonance, causes the scattering of the *y*-polarized incident THz wave into *x*-polarized light *t*_*yx*_. For the structure parameters identical to the Fig. 2(b), *t*_*yx*_ and *t*_*yy*_ are shown in the Fig. 6 with the Fermi level *E*_*f*_ = 0.6 *eV*. It can be seen that strong polarization conversion can be achieved at the two transmission dips at 2.3 THz and 2.9 THz.

**Figure 7:**
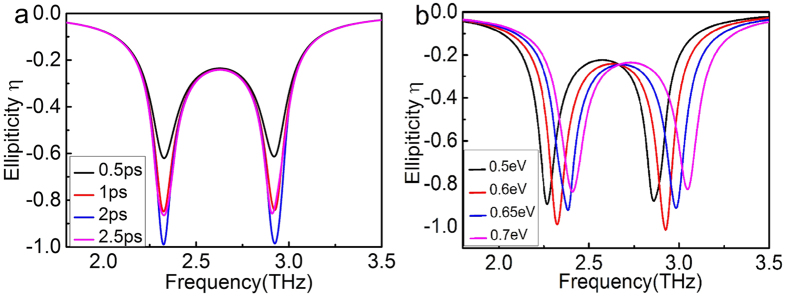
The ellipticity of transmitted THz wave at different (**a**) the relaxation time_*τ*_; **(b)** and the Fermi level *E*_*f*_.

Due to the phase difference between the two transmitted THz wave depending on the degree of coupling and the relaxation time^22^, the ellipticity of the transmission THz wave can be dynamically tuned. To systematically study the polarization state of the transmitted light, the ellipticity can be calculated by


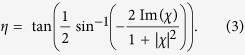


here, *χ* = *t*_*yx*_/*t*_*yy*_ is the complex-valued ratio of the *x*-polarized and *y*-polarized transmission coefficients. *η* = 0 corresponds to the linear *y*-polarized THz wave whereas *η* = −1 corresponds to the right circular polarization^31^. The increasing of relaxation time, resulting from changing the surrounding environment such as placing organic molecules on graphene^32^, leads to the change of the ellipticity. Figure 7(a) illustrates the ellipticity of transmitted THz wave at different *τ* with the other parameters same as those used in Fig. 2(b). It can be seen that the ellipticities of the two transmission dips decreases at first and then increases as *τ* increases. The reasons are that the charge carriers contributed to the graphene dipole and the monopole resonance increase with the relaxation time increasing, leading to the increasing of the coupling strength. Then, the total transmission enhances and the transmitted THz wave convert from elliptically polarization to right circular polarization. As *τ* continues to increase, the magnitude of the ellipticity decreases while the total transmission still enhance. Figure 7(b) demonstrates that the ellipticity of transmitted THz wave at different Fermi level with the relaxation time *τ* = 2 *ps*. It has been implied that the ellipticity can be actively tuned by varying the Fermi level of the two connected T-shape graphene strips. Therefore, the PIPC structure can be employed for realizing not only the polarization encoding but also polarization-division multiplexing (PDM), leading to impacting a wide range of photonic applications, ranging from optical communication to information encryption^25^.

## Discussion

In summary, the PIPC structure, composed of patterned graphene strips, not only has a transparency window with two optical enhanced transmittance dips for the co-transmissivity THz wave but also has the ability to convert the elliptically polarization to right circular polarization. Moreover, the peak frequency and the group index in the transparency window can be tuned by varying the Fermi level of the two connected T-shape graphene strips. Meanwhile, the coupled Lorentz oscillator model is applied to explain the formation mechanism of PIT based on graphene, with analytical fitting results in good agreement with the numerical simulations. Another characteristic of the PIPC structure is that the ellipticity can be tuned with changing the relaxation time and the Fermi level of the two connected T-shape graphene strips.

## Method

In our simulation, our method mainly focuses on the material effect in 2D flat surface while ignoring that in out-of-plane direction, resulting in regarding the graphene layer as an anisotropic material with in-plane conductivity *σ*^25^. At the room temperature and low THz frequency, the graphene’s complex surface conductivity can be described as 
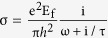
 within the local random phase approximation^33^,^34^. In the COMSOL Multiphysics, the graphene can be considered as surface current boundary condition^35^,^36^. The intrinsic relaxation time *τ* = 0.5 *ps* and the Fermi level *E*_f_ = 0.6 *eV* are initially considered and their influences would be analyzed later. And the Fermi level of two disconnected vertical graphene strips is fixed at 0.6 eV. These PIPC models are used in the finite element program COMSOL Multiphysics to carry out the simulations of electromagnetic wave interactions. The results of these simulations are presented in the previous sections. These simulation results can also be predicted by the Coupled Lorentz Oscillator model.

## Additional Information

**How to cite this article**: Ling, F. *et al*. Active tunable plasmonically induced polarization conversion in the THz regime. *Sci. Rep*. **6**, 34994; doi: 10.1038/srep34994 (2016).
